# Putative *Riemerella anatipestifer* Outer Membrane Protein H Affects Virulence

**DOI:** 10.3389/fmicb.2021.708225

**Published:** 2021-09-20

**Authors:** Qun Gao, Shuwei Lu, Mingshu Wang, Renyong Jia, Shun Chen, Dekang Zhu, Mafeng Liu, Xinxin Zhao, Qiao Yang, Ying Wu, Shaqiu Zhang, Juan Huang, Sai Mao, Xumin Ou, Di Sun, Bin Tian, Anchun Cheng

**Affiliations:** ^1^Research Center of Avian Diseases, College of Veterinary Medicine, Sichuan Agricultural University, Chengdu, China; ^2^Institute of Preventive Veterinary Medicine, Sichuan Agricultural University, Chengdu, China; ^3^Key Laboratory of Animal Disease and Human Health of Sichuan Province, Chengdu, China

**Keywords:** outer membrane protein, virulence factor, OmpH, B739_0832, *Riemerella anatipestifer*

## Abstract

*Riemerella anatipestifer* causes serious contagious disease in ducks, geese, and other fowl. However, as a harmful pathogen causing significant economic losses in the poultry industry, *R. anatipestifer* is still poorly understood for its pathogenesis mechanisms. In a previous study, we developed an indirect ELISA method for detecting *R. anatipestifer* infection using B739_0832 protein, a putative outer membrane protein H (OmpH) that is conserved among different serotypes of *R. anatipestifer*. Although OmpH in some pathogenic bacteria, such as *Pasteurella*, has been reported as a virulence factor, it is still not clear whether B739_0832 protein contributes to the virulence of *R. anatipestifer*. In this study, we confirmed that B739_0832 protein in *R. anatipestifer* localizes to the outer membrane. We constructed a *B739_0832* deletion mutant strain (Δ*B739_0832*) and assayed various effects from the deletion of B739_0832. Δ*B739_*0832 strain had a similar growth rate to wild-type *R. anatipestifer* CH-1. However, the survival rate of ducklings in 10 days after infection from Δ*B739_*0832 strain was 50%, whereas no ducklings survived from wild-type *R. anatipestifer* infection. Furthermore, the median lethal dose (LD_50_) of the Δ*B739_*0832 strain was approximately 150 times higher than that of the wild-type strain. Pathology examinations on infected ducklings found that, at 36 h after infection, bacterial loads in blood, liver, and brain tissues from Δ*B739_*0832-infected ducklings were considerably lower than those from wild-type infected ducklings. These results demonstrate that the B739_0832 protein contributes to the virulence of *R. anatipestifer* CH-1.

## Introduction

*Riemerella anatipestifer* is a Gram-negative, rod-shaped bacterium in the *Flavobacteriaceae* family, *Riemerella* genus (Segers et al., 1993). *Riemerella anatipestifer* is one of the most serious bacterial threats harming mostly the duck industry, but *R. anatipestifer* infection has also been reported in other waterfowl worldwide, causing heavy economic losses (Wang et al., 2010; Hu et al., 2012). *Riemerella anatipestifer* infection has high mortality and morbidity rate in young ducklings, but may be present in adult ducks causing only subclinical or even asymptomatic diseases (Chang et al., 2019), making it difficult to detect and eradicate. There are at least 21 serotypes of *R. anatipestifer* reported around the world, with serotypes 1, 2, and 10 being responsible for most of the major outbreaks in China (Pathanasophon et al., 2002; Hu et al., 2012; Wang et al., 2014). As an important worldwide poultry pathogen, its exact pathogenic mechanism is still not clear. There is no cross-protection between different serotypes of *R. anatipestifer* developed from infection (Kang et al., 2018). With the diverse serotype variations, vaccines developed against *R. anatipestifer* have been shown to have low cross-protection against different serotypes (Chu et al., 2015). Antibiotics have been used to control *R. anatipestifer* infection in ducks. However, increasing evidence of drug resistance bacteria and detection of antibiotics in meat product call for safer alternatives. To safely and effectively control and prevent diseases caused by *R. anatipestifer*, new strategies for developing vaccines with cross-protection against different serotypes are needed.

Many outer membrane proteins in pathogenic bacteria are virulence factors that enable or facilitate bacterial attachment to host cell surfaces, as part of the pathogenicity process (Caruana and Walper, 2020). Outer membrane proteins of pathogenic bacteria are generally immunogenic and play important roles in virulence and immunity to bacterial diseases (Weiser and Gotschlich, 1991; Hatfaludi et al., 2010). To date, few virulence-associated proteins have been reported in *R. anatipestifer*, such as outer membrane protein A (OmpA), CAMP cohemolysin, Cas9, and TonB-dependent receptor (Crasta et al., 2002; Hu et al., 2011; Liao et al., 2015; Wang et al., 2019; Xu et al., 2020). Outer membrane protein H (OmpH) is a major outer membrane protein, conserved in Gram-negative bacteria (Hatfaludi et al., 2010). OmpH has been extensively studied in *Pasteurella multocida* as an immunodominant porin protein (Kim et al., 2011) and has been reported as an important virulence factor and a protective antigen for developing vaccine against *Pasteurellosis* in chickens (Thanasarasakulpong et al., 2015). Meanwhile, OmpH (also known as Skp) in *Escherichia coli* and *Salmonella typhimurium* has been identified as a periplasmic chaperone protein with Sec-A like activity and is involved in the maintenance of folding intermediates of outer membrane proteins (Schäfer et al., 1999).

In *R. anatipestifer* CH-1, B739_0832 open reading frame (ORF) is annotated as encoding an OmpH protein (Wang et al., 2014). In our previous study, we successfully developed an indirect ELISA assay using a recombinant B739_0832 protein for detecting *R. anatipestifer* infection (Gao et al., 2016). The B739_0832-based ELISA assay has higher sensitivity and wider detection range than OmpA-based ELISA and the conventional tube agglutination assay, suggesting that B739_0832 is conserved among different *R. anatipestifer* serotypes. To assess the roles of B739_0832 in *R. anatipestifer* pathogenesis, we evaluated effects of B739_0832 deletion on *R. anatipestifer* virulence.

## Materials and Methods

### Bacterial Strains, Plasmids, and Growth Conditions

Bacterial strains, plasmids, and primers used in this study are listed in Tables 1, 2, respectively. *Riemerella anatipestifer* CH-1 (RA CH-1) strain was used as the wild-type strain, and all other strains used were derived from RA CH-1. *R. anatipestifer* was grown in Tryptic Soy Broth (TSB) or Giolitti-Cantoni Broth (GCB) at 37°C with shaking (Liu et al., 2017). GCB agar plates were prepared by supplementing GCB with 1.5% agar. Alternatively, *R. anatipestifer* was also grown on Luria-Bertani (LB) agar plate supplemented with 5% sheep blood. When necessary, appropriate concentrations of antibiotics were added to the media: ampicillin (Amp, Sigma-Aldrich, 100 μg/ml), chloramphenicol (Cm, Sigma-Aldrich, 30 μg/ml), kanamycin (Kan, Sigma-Aldrich, 50 μg/ml), and cefoxitin (Cfx, Sigma-Aldrich, 1 μg/ml).

**TABLE 1:** Strains and plasmids used in this study.

Strains	Genotype or description	Source or references
*Riemerella anatipestifer* CH-1	Kan^R^	Laboratory collection
Δ*B739_0832*	*R. anatipestifer* CH-1 Δ*B739_0832*	This study
CΔ*B739_0832*	*R. anatipestifer* CH-1 Δ*B739_0832* carrying pLMF02:*B739*_*0832* plasmid	This study

**Plasmids**	**Genotype or description**	**Source or references**

pLMF02	Derivative of pPM5, Amp^R^, Cfx^R^	Liu et al., 2017
pLMF02:*sacB*	Derivative of pLM02, Amp^R^, Km^R^, Cfx^R^	Tian et al., 2020
pLMF02:*B739_0832*	pLMF02 carrying *B739_0832* from *R. anatipestifer* CH-1, Cfx^R^	This study

*Amp^R^, ampicillin resistance; Kan^R^, kanamycin resistance; Cfx^R^, cefoxitin resistance.*

**TABLE 2:** Primers used in this study.

Primers	Sequence (5′–3′)	Organism or references
16S rRNA P1	CGAAAGTGATAAGTTAGCCACCT	Zhang et al., 2017
16S rRNA P2	GCAGCACCTTGAAAATTGTCC	
*B739_0831* P1	CTCAATACAAAGAGGCAGAA	This study
*B739_0831* P2	TTCCCTTGTCTTTAGTTGCT	
*B739_0833* P1	GCGACCCAATAGGGCATC	
*B739_0833* P2	GGTAAATCCGTAGTTATCTTCCAC	
Δ*B739_0832* uparm P1	CTAGCTAGCCGACTTTGCTTACGGATTTG	
Δ*B739_0832* uparm P2	GGGGTACCAATAATAAATAAAGTTTAATTTTTATAGTTTTTTATT	
Δ*B739_0832* downarm P1	ACGCGTCGACTTAAGTTGAAAATATCTATAAAGCCAC	
Δ*B739_0832* downarm P2	AACTGCAGCAATTACCTAATTGTCCCCCTGC	
Δ*B739_0832* overlap uparm P1	CTTTGCTTACGGATTTGATAAAACTATAG	
Δ*B739_0832* overlap uparm P2	GCTTTATAGATATTTTCAACTTAAAATAATAAATAAAGTTTAATTTTTATAG	
Δ*B739_0832* overlap downarm P1	TAAAAATTAAACTTTATTTATTATTTTAAGTTGAAAATATCTATAAAGCCAC	
Δ*B739_0832* overlap downarm P2	TTACCTAATTGTCCCCCTGCAC	

### Construction of Clean *B739_0832* Deletion Mutant Strain (Δ*B739_0832*)

A Δ*B739_0832* mutant strain was constructed by using the natural transformation method as previously described (Liu et al., 2017). Briefly, about 700-bp fragments upstream and downstream of *B739_0832* gene were amplified using primer pairs Δ*B739_0832* up-arm P1 and P2, Δ*B739_0832* down-arm P1 and P2, respectively (Table 2). The amplified upstream and downstream fragments were connected to a pLMF02:sacB plasmid using ligation (Tian et al., 2020). The recombined plasmid was further processed with restriction enzymes *Nhe*I and *Pst*I to extract a DNA fragment that has a Cfx-sacB cassette in the center flanked by the amplified upstream and downstream fragments. The DNA fragment was purified using a Universal DNA Purification kit (TIANGEN^TM^, Beijing, China) and served as donor DNA. Wild-type *R. anatipestifer* CH-1 was transformed with the purified DNA fragment, and cefoxitin-resistant, sucrose-sensitive recombinants were scored. Another DNA fragment fusing the upstream and downstream fragments were produced using the overlap PCR method. The scored cefoxitin-resistant, sucrose-sensitive recombinant strain was further transformed with the upstream–downstream overlap DNA fragment; the Δ*B739_0832* mutant strain was scored as cefoxitin-sensitive and sucrose-resistant recombinants. The Δ*B739_0832* mutant strain was verified by PCR amplification and sequencing (Supplementary Figure 1).

A complementary strain (CΔ*B739_0832*) was prepared by transforming the Δ*B739_0832* mutant strain with a B739_0832 expressing plasmid (pLMF02:*B739_0832*). Briefly, B739_0832 gene was cloned into pLMF02—a pPM5 derivative—plasmid (Tian et al., 2020). The Δ*B739_0832* mutant strain was then transformed with the pLMF02:*B739_0832* plasmid using the natural transformation method as described in Liu et al. (2017) and selected for cefoxitin resistance colonies. Expression of B739_0832 in the complemented strain was confirmed by Western blot.

### Quantitative Real-Time PCR

Quantitative real-time PCR (qRT-PCR) was used to measure transcription expression levels of *B739_0832* and flanking genes (Liu et al., 2016). Total RNA of the wild-type strain and the Δ*B739_0832* mutant strain was extracted from cell cultures at OD_600_ = 1.0 using RNAprep pure Cell/Bacteria kits (TIANGEN^TM^, Beijing, China). To eliminate DNA contamination, all extracted total RNA samples were treated with RNase-free DNase I (40 U/mg RNA, Takara, China) and purified using RNeasy Mini Kits (Qiagen, Germany). HiScript reverse transcriptase (Vazyme, China) was used to generate cDNA in accordance with manufacturer’s instructions. qRT-PCR was performed using SYBR Green Master Mix (Bio-Rad, United States) and primers listed in Table 2. The expression level of 16S rRNA was used as an internal control. Measurements were performed with three separate cell samples for each gene and were replicated in triplicate. Data were analyzed with a normalized gene expression method (2^–ΔΔCt^) as previously described (Pfaffl, 2001).

### Growth Rate Determination

Bacterial growth rates were determined as previously described (Luo et al., 2015). Briefly, each strain was activated on an LB plate supplemented with 5% sheep blood overnight at 37°C. A single colony from each strain was inoculated into 5 ml of TSB and cultured at 37°C with agitation for 10 h. Subsequently, each culture was adjusted to an OD_600_ of 0.05 in 20 ml of fresh TSB and grown at 37°C with shaking at 180 rpm. OD_600_ for each culture was determined at every 1 h for 18 h.

### Total Membrane Extraction, Separation of Inner Membrane and Outer Membrane, and Western Blot

*Riemerella anatipestifer* total membrane, inner membrane, and outer membrane were extracted and separated based on methods previously described by Hu D. et al. (2019), Thein et al. (2010), and Osborn and Munson (1974). Cells were grown in 1 L TSB to OD_600_ ≈ 3 at 37°C. Chloramphenicol was then added to the culture at 1 mg/ml final concentration to stop protein synthesis and cells with chloramphenicol were agitated for another hour to ensure that all localization processes are completed. Cells were harvested and resuspended in 10 ml of 0.2 M Tris–HCl, pH 8.0, 1 M sucrose, and 1 mM EDTA, and lysozyme was added to a final concentration of 1 mg/ml. The cells were incubated on ice for 10 min. Spheroplast was prepared by slowly mixing 40 ml ice-cold H_2_O into the cell suspension. The cells were collected by centrifugation at 200,000 × *g* for 45 min at 4°C. The cell pellet was resuspended in 10 ml of ice-cold 10 mM Tris–HCl, pH 7.5, 5 mM EDTA, 0.2 mM DTT, and 1 mg/ml DNase. The cells were lysed by passing through a French Press twice at 10^8^ Pa. The sample after French Press was centrifuged at ∼3,000 × *g* for 15 min to remove cell debris. The supernatant was ultracentrifuged at 120,000 × *g* for 2 h at 4°C to collect total membrane. The total membrane pellet was resuspended in 1 ml of ice-cold 10 mM Tris–HCl, pH 7.5, 15% sucrose (w/v), 5 mM EDTA, and 0.2 mM DTT. Inner membrane and outer membrane were separated on a sucrose step gradient (1 ml of 55% sucrose and 2.25 ml each of 50, 45, 35, and 30% sucrose). The total membrane suspension was placed on top of the sucrose gradient and centrifuged at 250,000 × *g* for 12 h at 4°C. The outer membrane (lower band, higher density) and inner membrane (upper band, lower density) were extracted by syringes. Each sample was washed three times with 1 ml of Tris buffer (10 mM Tris–HCl, pH 7.5, and 1 mM EDTA) and centrifuged at 100,000 × *g* for 20 min to collect the membrane samples. The samples were resuspended in SDS-PAGE sample loading buffer for analysis. Whole cell samples were prepared by collecting 2 ml of cells and resuspended in 1 ml of SDS-PAGE sample loading buffer.

His-tagged B739_0832 expression plasmid [pET32a(+)-ompH] was obtained in our previous study (Gao et al., 2016). His-tagged B739_0832 protein was purified and used to generate rabbit polyclonal antibody, as described before (Zhang et al., 2017). Whole cell, total membrane, inner membrane, and outer membrane of *R. anatipestifer* samples were analyzed using Western blot assay; OmpA was used as an outer membrane protein reference, TonB was used as an inner membrane protein reference, and RecA was used as a cytoplasmic protein reference (Thein et al., 2010; Liao et al., 2015; Xu et al., 2020).

### Assessment of LD_50_

Groups of 3-day-old Cherry Valley ducklings were used to assess LD_50_ of Δ*B739_0832* and wild-type strains. Ducklings were divided into 16 groups (10 ducklings per group, 160 total): 5 groups were challenged with wild-type *R. anatipestifer* CH-1, 5 groups with Δ*B739_0832*, 5 groups with CΔ*B739_0832*, and 1 group with saline control. The wild-type group was intramuscularly injected at a dose of 10^6^ to 10^10^ CFU; the Δ*B739_0832* group was injected at a dose of 10^7^ to 10^11^ CFU, the CΔ*B739_0832* group was injected at a dose of 10^6^ to 10^10^ CFU, and the control group was injected with 1 ml of sterile phosphate-buffered saline (PBS). LD_50_ values were calculated using SPSS 23.0 (Arambašic and Randhawa, 2014).

### Determination of Bacterial Load in Infected Duck Tissues

Three groups of 3-day-old Cherry Valley ducklings (15 ducklings per group) were intramuscularly injected with wild-type, Δ*B739_0832*, or CΔ*B739_0832* at a dose of 10^9^ CFU, respectively. After challenge, blood, liver, heart, brain, and spleen tissues samples were collected at 6, 12, 24, 36, 48, and 72 h. Three ducklings were randomly selected for sacrifice at each time point. The organ samples were weighed and transferred into tubes each containing 3 ml of sterilized PBS. After homogenization, the tubes were centrifuged for 5 min at 2,000 × *g* to remove cell debris, the supernatant of each tube was serially diluted with PBS, and 50 μl of each serial dilution was plated on a TSA plate. TSA plates were incubated at 37°C overnight for bacterial count.

### Assessment of Duck Survival Rate

Forty 3-day-old Cherry Valley ducklings were randomly divided into four groups (10 ducks per group). One group was challenged with a dose of 10^10^ CFU wild type, one with Δ*B739_0832*, one with CΔ*B739_0832*, and the fourth group as a control was intramuscularly injected with equal volume (1 ml) PBS. The ducklings were observed for 10 consecutive days after the challenge. All ducklings were indoor and had access to plenty of food and water. Survival rates were calculated each day as the proportion of living ducklings accounted for the initial duckling counts.

### Bacterial Adhesion Assay

Bacterial adhesion assay was performed with duck embryo fibroblast (DEF) cells as previously described (Hu et al., 2011). Briefly, each well in a 24-well tissue culture plate was seeded with 1 ml of 2 × 10^5^ cells/ml DEF cells in Dulbecco’s Modified Eagle Medium (DMEM; Biowest, France) and incubated at 37°C with 5% CO_2_ for 18 h. After confirming that there was at least 95% confluence and has no contamination, each well was infected with 10^7^ CFU *R. anatipestifer* (multiplicity of infection MOI = 50:1). The plates were then incubated at 37°C with 5% CO_2_ for another 1.5 h. After incubation, the wells were washed three times with PBS to remove non-adherent bacteria and then incubated at 37°C with 5% CO_2_ for 10 min in the presence of 0.25% trypsin (100 μl/well) to release the DEF cells from the wells. Serial 10-fold dilutions were prepared from the cell suspensions and 50 μl of each dilution was plated onto TSA plates to determine adhered bacteria counts. Each assay was performed in triplicate and replicated three times.

### Bacterial Invasion Assay

Bacterial invasion assay was also performed with DEF cells as previously described (Hu et al., 2011). DEF cells were grown in 24-well tissue culture plates and then infected with *R. anatipestifer* the same way as the adhesion assay described above. For the invasion assay, after the infection incubation, 100 μg/ml gentamicin was added to each well and the plate was incubated for an additional 1 h at 37°C to kill all extracellular bacteria. After the extra incubation, the wells were washed three times with PBS and treated with 100 μl of 1% Triton X-100 to lyse the DEF cells. Lysed cells were homogenized. Serial 10-fold dilutions were prepared from the cell lysate and 50 μl of each dilution was plated onto TSA plates to determine invasive bacteria counts. Each assay was performed in triplicate and replicated three times.

### Statistical Analysis and Ethics Statement

Statistical analysis was performed with GraphPad Prism 7.0 for Windows (GraphPad Software Inc., San Diego, CA, United States) (Hu Y. C. et al., 2019). Significance of difference between two data sets was evaluated using Student’s *t*-test, and a value of *p* < 0.05 was considered significant (Mishra et al., 2019).

Three-day-old Cherry Valley ducklings were procured from Sichuan Agricultural University duck farm and kept under appropriate conditions with a 12-h light/dark cycle and free access to food and water during this study. All ducks were handled in strict adherence to the recommendations of the local animal welfare bodies and Sichuan Agricultural University (No. XF2014-18). The animal-use procedures were approved by the Animal Ethics Committee of Sichuan Agricultural University (Approval No. 2016-015).

## Results

### Bioinformatics Analysis of B739_0832 Locus in RA CH-1 Strain

In the National Center for Biotechnology Information (NCBI) database, the *B739_0832* locus in RA CH-1 has been identified as a 501-base-pair ORF, which encodes a 166-amino acid protein, with a molecular mass of about 18 kDa. The B739_0832 protein has been annotated as an OmpH family outer membrane protein. We analyzed the amino acid sequences of B739_0832 proteins from all sequenced *R. anatipestifer* strains using the protein–protein Basic Local Alignment Search Tool (BLASTP). The sequence alignment results from BLASTP indicated over 95% identity among different *R. anatipestifer* strains.

OmpH (also known as Skp) proteins in some Gram-negative bacteria have been shown to be either outer membrane protein or chaperone proteins for outer membrane proteins. We compared the amino acid sequence of B739_0832 protein from *R. anatipestifer* strains with the OmpH (Skp) sequences from *E. coli*, *Salmonella*, and *Pasteurella* using Clustal Omega, a multiple sequence alignment tool from EMBL-EBI (Madeira et al., 2019; Supplementary Figure 2). The amino acid sequence alignment results indicated that the B739_0832 protein is closer in evolution to OmpH from *Pasteurella* than those from *E*. *coli* or *Salmonella*. We also analyzed the hydrophobicity properties of the B739_0832 protein using ExPASY software from SIB Swiss Institute of Bioinformatics (results not shown). The results indicated that, of the 166 residues in B739_0832 protein, 63 of them are hydrophobic, and both carboxyl and amino ends of the protein show more hydrophobicity than the middle portion. The results are consistent with outer membrane protein propensities.

### Construction and Characterization of Δ*B739_0832* Strain and Complemented Strain CΔ*B739_0832*

To elucidate functions of *B739_0832* in RA CH-1, we constructed a *B739_0832* clean deletion strain (Δ*B739_0832*) and a complemented strain (CΔ*B739_0832*) using the natural transformation method as described in “Materials and Methods.” Deletion of the *B739_0832* gene was confirmed by PCR amplification using primers flanking the locus. PCR amplification of the 16S rRNA gene was performed at the same time as a positive control. The confirmed *B739_0832* deletion mutant strain was named as Δ*B739_0832*. The *B739_0832* gene from the RA CH-1 wild-type strain was amplified and cloned into a pLM02 vector plasmid to generate a recombination plasmid pLMF02:*B739_0832*. This recombination plasmid was introduced into the Δ*B739_0832* strain by conjugation to yield the complemented strain CΔ*B739_0832*.

**FIGURE 1:**
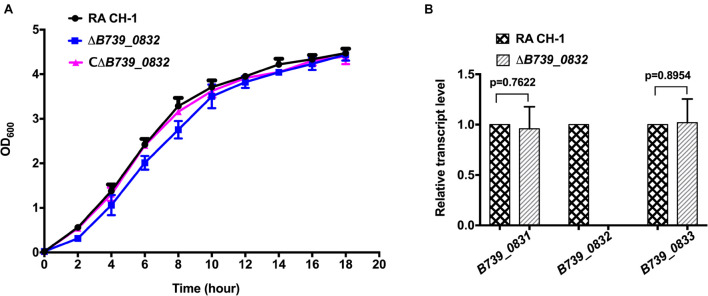
Characterization of mutant strain Δ*B739_0832* and complemented strain CΔ*B739_0832*. **(A)** Growth curves of *Riemerella anatipestifer* CH-1, Δ*B739_0832*, and CΔ*B739_0832* strains in Tryptic Soy Broth (TSB). Cells were inoculated in 25 ml of fresh TSB at 37°C with an initial OD_600_ of 0.05. OD_600_ values for each culture were subsequently measured every 2 h for 18 h. Data represent the mean values of three experiments. **(B)** Gene transcription levels in Δ*B739_0832* strain were analyzed using quantitative PCR (qPCR). Transcription levels of *B739_0832* and the flanking genes *B739_0831* and *B739_0833* in *R. anatipestifer* CH-1 and Δ*B739_0832* strains were measured. Expression of *B739_0832* was completely inactivated in the Δ*B739_0832* mutant strain. Expression of upstream gene *B739_0831* and downstream gene *B739_0833* had no significant difference compared to wild type. Data were analyzed using Student’s *t*-test. Error bars represent standard deviations of three independent repeats.

We compared the growth rates of wild-type RA CH-1, ΔB739_0832, and CΔB739_0832 strains. The growth rate of the mutant strain ΔB739_0832 showed no significant difference from that of the wild-type strain and the complemented strain (Figure 1A). We further tested transcription levels of genes upstream (*B739_0831*) and downstream (*B739_0833*) of the *B739_0832* locus. As shown in Figure 1B, the transcription levels of upstream and downstream genes were not affected by the deletion of *B739_0832*, which indicates that deletion of *B739_0832* did not cause polar effect.

### B739_0832 Protein Is an Outer Membrane-Associated Protein

**FIGURE 2:**
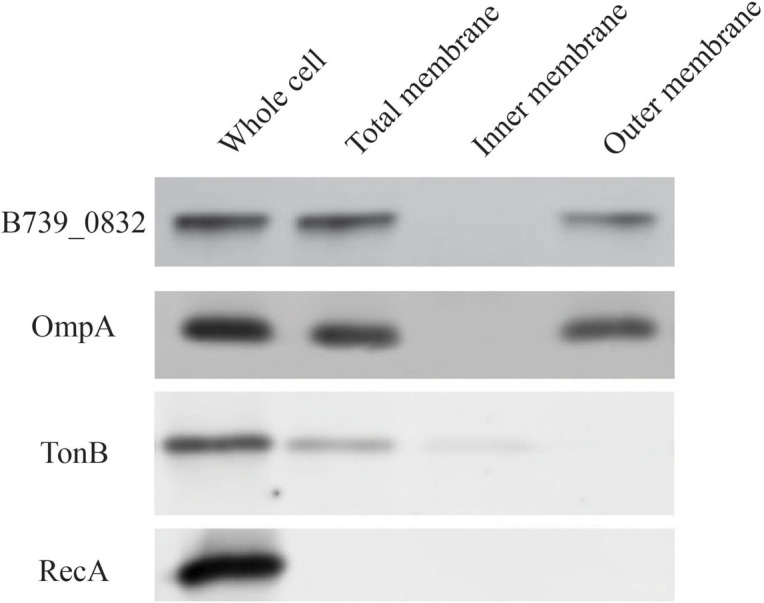
Membrane localization of *R*. *anatipestifer* CH-1 B739_0832 protein. Isolated subcellular fractions of *R. anatipestifer* cells were prepared as described in “Materials and Methods” and analyzed using Western blot. Results indicate that B739_0832 protein localizes in the outer membrane. OmpA is a confirmed outer membrane protein in *R. anatipestifer*, RecA is a known cytoplasmic protein and TonB is a known inner membrane protein. See Supplementary Figure 3 in the Supplementary Material for more information.

To determine localization of B739_0832 protein in *R. anatipestifer* cells, we isolated inner membrane, outer membrane, and total membrane subcellular fractions in RA CH-1 cells and compared the localization of B739_0832 to known outer membrane protein OmpA (outer membrane porin protein), known inner membrane protein TonB (energy transducer), and known cytoplasmic protein RecA (DNA maintenance and repair protein; Figure 2). In order to avoid false positive caused by proteins during transport, we added 1 mg/ml chloramphenicol to stop protein synthesis and incubated the bacterial cells for another 40 min to ensure all fully synthesized proteins are transported to their final destinations. B739_0832 protein was detected in outer membrane fraction, similar to OmpA, but not in inner membrane fraction. The localization data are consistent with the bioinformatics data that B739_0832 is more closely related in evolution to OmpH from *Pasteurella* than OmpH (Skp) from *E*. *coli*. It is also consistent with our previous ELISA study that B739_0832 is an exposed antigen in live *R. anatipestifer* cells.

### Δ*B739_0832-*Infected Ducklings Have Increased Survival Rate Than Those Infected by Wild Type

**FIGURE 3:**
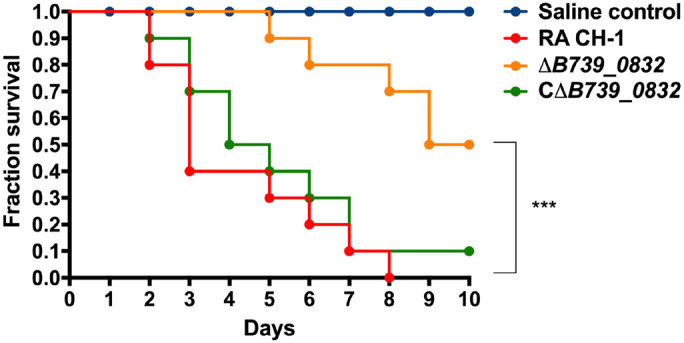
Survival rate in ducklings infected by *R. anatipestifer* CH-1, Δ*B739_0832*, or *C*Δ*B739_0832* strains. Each group has 10 3-day-old ducklings, which was injected intramuscularly at a dose of 10^10^ CFU to assess the survival rate. The group shown in red was injected with wild-type *R. anatipestifer* CH-1. The orange group was injected with Δ*B739_0832*. The green was injected with CΔ*B739_0832*. Blue represents the control injected with phosphate-buffered saline (PBS). Data were analyzed using a Log-rank (Mantel–Cox) test. Three stars indicate significant difference (*p* < 0.001).

To determine the impact of *B739_0832* deletion on RA CH-1 virulence, we measured the mortality rates in ducklings caused by RA CH-1, Δ*B739_0832*, and CΔ*B739_0832* strains. Groups of 3-day-old Cherry Valley ducklings were infected by one of these three strains at a dose of 10^10^ CFU and were observed for 10 days. At day 7 after infection, ducklings infected by Δ*B739_0832* had about 70% survival rate, whereas ducklings infected by wild-type RA CH-1 or complemented strain CΔ*B739_0832* only had about 10% survival rate. After 10 days, no ducklings survived from infection by RA CH-1, and only 10% survived infection from CΔ*B739_0832*, whereas 50% ducklings survived from infection by Δ*B739_0832*. The wild-type and complementary groups had similar patterns; about half of the ducklings in these two groups did not survive for more than 5 days. However, survival rates in the Δ*B739_0832* group were significantly different from those in the wild-type and complementary groups (Figure 3).

To further quantify the impact of *B739_0832* deletion on RA CH-1 virulence, we measured half lethal dose (LD_50_) of these three strains. The LD_50_ of RA CH-1 was 3.98 × 10^8^, which was about 150 times lower than that of the Δ*B739_0832* strain (6.09 × 10^10^). The LD_50_ of complemented strain CΔ*B739_0832* was 7.76 × 10^8^, which was similar to wild type.

### Deletion of *B739_0832* Gene Decreased *Riemerella anatipestifer* Adhesion and Invasion in Duck Embryo Fibroblast Cells

**FIGURE 4:**
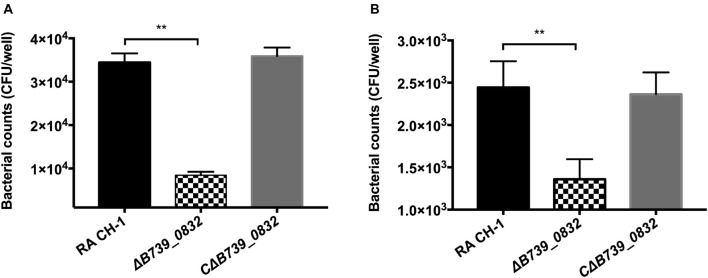
Adherence and invasion assays for *R. anatipestifer* CH-1, Δ*B739_0832*, and CΔ*B739_0832* strains. Both adherence and invasion assay were performed using duck embryo fibroblast (DEF) cells. **(A)** Bacteria count from adherence assay measurements. Δ*B739_0832* showed significantly lower bacterial count, but maintained some level of host cell adherence ability. **(B)** Measurements of bacterial invasion ability. Δ*B739_0832* showed significantly lower host cell invasion ability, but did not completely abolish its invasion ability. All data were analyzed with Student’s *t*-test. Error bars represent standard deviation from three sets of independent measurements (**p < 0.01).

To assess whether deletion of *B739_0832* gene affected adherence and invasion activities of *R. anatipestifer*, the activities of wild-type, Δ*B739_0832*, and CΔ*B739_0832* strains were measured using DEF cells. DEF cells were infected at MOI of 50:1; the Δ*B739_0832* strain had 8.77 ± 1.17 × 10^3^ CFU/well adhesion activity, which was approximately threefold lower than that of wild type (3.44 ± 0.16 × 10^4^ CFU/well; Figure 4A). Bacterial invasion tests were performed under similar testing conditions. After killing all extracellular bacteria by gentamicin, bacterial counts inside host cells infected by Δ*B739_0832* strain were 1.36 ± 0.1 × 10^3^ CFU/well, which was approximately twofold lower than those infected by wild type (2.44 ± 0.2 × 10^3^; Figure 4B). The complemented strain CΔ*B739_0832* had almost identical activities as wild type for both adhesion and invasion tests.

### Deletion of *B739_0832* Gene Attenuated *Riemerella anatipestifer* Virulence

**FIGURE 5:**
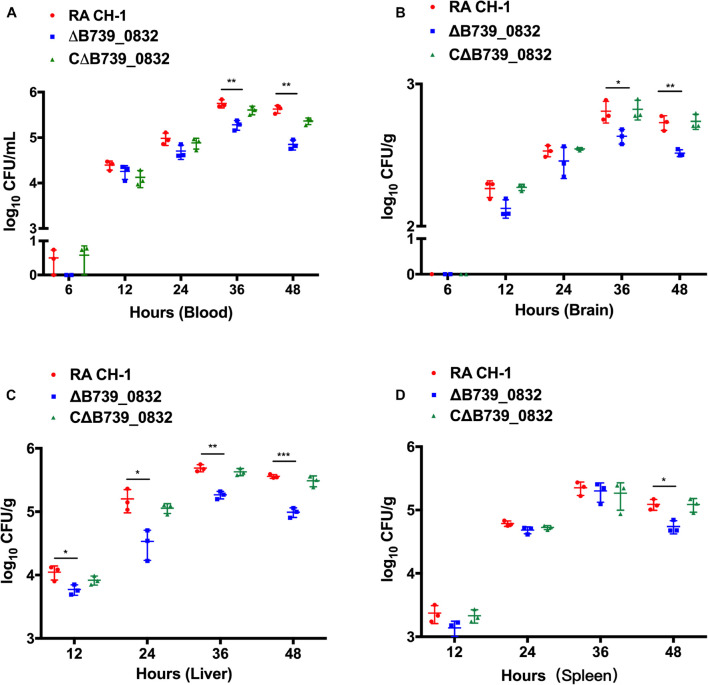
Bacterial loads in blood **(A)**, brain **(B)**, liver **(C)**, and spleen **(D)** of ducks infected with *R. anatipestifer* CH-1, Δ*B739_0832*, or CΔ*B739_0832* strains at 6, 12, 24, 36, and 48 h post-infection. Error bars represent standard deviations from three independent experiments (***p < 0.001, **p < 0.01, and *p < 0.05).

To further evaluate the influence of Δ*B739_0832* on systemic infection *in vivo*, bacterial loads in blood, liver, spleen, and brain from ducks infected by wild type, Δ*B739_0832*, or CΔ*B739_0832* were quantified. Bacterial loads from all three groups were almost identical for the first 24 h after infection, with the exception of bacterial loads in liver. However, a difference slowly developed at 36 and 48 h (Figures 5A–D). In brain and blood, the difference developed at 36 h, earlier than in spleen, which did not show significant difference until 48 h, whereas, in liver, the bacterial loads were different since 12 h post-infection, and the difference grew more significant at 48 h (Figure 5C).

**FIGURE 6:**
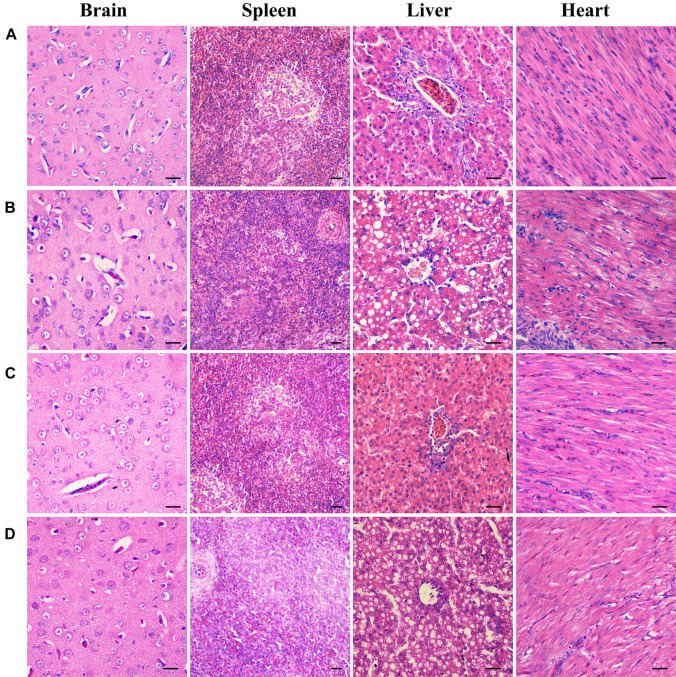
Organ histopathological changes in ducks infected by *R. anatipestifer*. **(A)** Brain, spleen, liver, and heart samples from a healthy duck. **(B)** Corresponding samples from ducks infected by wild-type *R. anatipestifer* CH-1 at 36 h post-infection. **(C)** Corresponding samples from ducks infected by mutant strain Δ*B739_0832* at 36 h post-infection. **(D)** Corresponding samples from ducks infected by complement strain CΔ*B739_0832* at 36 h post-infection. For both brain and spleen tissues, samples from all three strains showed no lesions. For *R. anatipestifer* CH-1-infected ducks, obvious pathological changes were observed in liver and heart: liver cord disorder, a large area of vacuole-like changes and myocardial necrosis. Δ*B739_0832* mutant strain-infected ducks showed no pathological changes in liver and heart. CΔ*B739_0832*-infected ducks had the same histopathological changes as the wild-type strain. Tissue samples were stained with hematoxylin and eosin (400×). The size of the scale bar is 20.0 μm.

To further examine the effects of deletion of B739_0832 gene on virulence, we compared organ tissue lesions in ducks infected by wild-type, Δ*B739_0832*, or CΔ*B739_0832* strains. At 36 h post-infection, we collected heart, liver, spleen, and brain tissue samples from groups of ducklings infected by each of the three strains for histopathological examination. The tissue samples were stained with hematoxylin and eosin to visualize lesions caused by *R. anatipestifer* invasion. All brain tissues exhibited no visible damage, suggesting that *R. anatipestifer* had not passed the blood–brain barrier yet at this time point. However, liver cord disorders, involving a large number of vacuole-like changes, were clearly visible in samples infected by wild-type and complementary strain CΔ*B739_0832*. Myocardial necrosis was also present in heart samples infected by wild-type and CΔ*B739_0832* strains. However, there were no visible lesions in liver or heart samples infected by the Δ*B739_0832* strain (Figure 6).

## Discussion

*Riemerella anatipestifer* can infect a variety of domestic and wild birds, such as ducks, geese, and turkeys. Therefore, it is important to understand the pathogenic mechanisms of *R. anatipestifer* for controlling its spread. It is well known that outer membrane proteins in Gram-negative bacteria play important roles in stimulation of host immune systems (Navidinia et al., 2019). OmpH has been demonstrated to be a major outer membrane protein present in *P. multocida* envelope (Lugtenberg et al., 1986). A study has shown that purified native OmpH protein from the *P. multocida* A:3 strain could be used to elicit immune responses providing homologous protection in chickens (Luo et al., 1997), supporting OmpH in *P. multocida* as an exposed outer membrane protein. However, OmpH in *E*. *coli* and *S*. *typhimurium*, also known as Skp, has been identified as a periplasmic chaperone protein, despite initial reports of it as DNA binding protein (Holck et al., 1987), or outer membrane protein (Koski et al., 1989).

Our data suggest that B739_0832 protein in *R. anatipestifer*, a putative OmpH protein, is probably an outer membrane protein, consistent with it being more closely related to OmpH in *P. multocida* than in *E*. *coli* or *S*. *typhimurium*. The localization data suggest that B739_0832 protein is probably outer membrane protein, or at least outer membrane associated due to the limitation of our methods (Figure 2). Bioinformatics analysis found that over one-third amino acids in B739_0832 protein are hydrophobic, further suggesting that B739_0832 protein could be a membrane-inserted protein. The impacts of *B739_0832* gene deletion on *R. anatipestifer* virulence illustrated in this study, combining our previous study in successfully developing B739_0832 protein-based ELISA method for detecting live *R. anatipestifer* cells, suggest that B739_0832 in *R. anatipestifer* is probably an outer membrane protein, or at least is a membrane-associated protein.

Studies have shown that OmpH proteins in both *P. multocida* and *E. coli* can associate with lipopolysaccharide (LPS; Luo et al., 1997; Bulieris et al., 2003). LPS, as a cell wall component characteristic of Gram-negative bacteria, is a pathogen-associated molecule that plays an important role in triggering bacteria-infected host innate immune responses. Luo et al. believed that it was OmpH protein, not the small amount LPS contaminant, that elicited immune response in turkeys, whereas, in *E. coli*, Skp protein is more involved in facilitating outer membrane proteins through the periplasmic region. Skp’s association with LPS may partly be due to the numerous positively charged amino residues distributed throughout the protein and probably has no direct role as an antigen. In this study, we found that B739_0832 affects *R. anatipestifer* virulence and is likely involved in host cell attachment and invasion. It is very possible, due to its similarity to OmpH proteins in *P. multocida* and *E. coli*, that B739_0832 in *R. anatipestifer* is also associated with LPS. However, it is not clear what role, if any, LPS plays in the virulence effects of B739_0832. The relationship and association between B739_0832 and LPS need to be studied further and will probably shed light on pathogenesis mechanisms of *R. anatipestifer*.

Our results show that B739_0832, although not required for growth, is heavily involved in *R. anatipestifer* virulence. Deletion of *B739_0832* gene greatly reduced its virulence: effecting lower mortality rate, higher survival rate in ducklings infected by Δ*B739_0832*, and much higher LD_50_. Δ*B739_0832* also showed attenuated pathogenic effects on host organs (no visible lesions found). However, only 50% of ducklings infected by Δ*B739_0832* survived for over 10 days, suggesting that B739_0832, although important for virulence, is not critical for pathogenesis. In fact, Δ*B739_0832* could still attach and invade DEF cells and was detected in all examined host organs, albeit at lower amount than wild type. These lines of evidence strongly suggest that B739_0832 is involved in the initial invasion process of *R. anatipestifer*. It could be part of the host cell attachment process, which further confirms that B739_0832 is an outer surface protein.

The striking differences between B739_0832 and *E*. *coli* Skp protein are underscored in their sequence differences. Charged residues, especially positively charged residues, in *E*. *coli* Skp have been shown to play important functional roles (Bulieris et al., 2003). However, B739_0832 has fairly different distribution of charged residues than *E*. *coli* Skp. For example, comparing residues 21–45, about one-third (eight residues) of the total 25 residues changed from neutral to charged residues in B739_0832. This type of asymmetric distribution of charged residues is often observed in outer membrane proteins (Slusky and Dunbrack, 2013). It appears that these two proteins diverged at some point during evolution: one became an outer membrane protein, whereas another became a chaperone for outer membrane protein. However, it is not clear which role was earlier in the evolution process.

In summary, our earlier study showed that B739_0832-based ELISA could be used to detect *R. anatipestifer* of different serotypes with high sensitivity. In this study, we provide further evidence that B739_0832 is an outer membrane protein. We also demonstrated, for the first time, that B739_0832 is involved in *R. anatipestifer* virulence. B739_0832 is highly conserved among different serotypes. Its involvement in virulence further supports that B739_0832 is a good candidate as a universal antigen for developing vaccine for all serotypes.

## Data Availability Statement

The original contributions presented in the study are included in the article/Supplementary Material, further inquiries can be directed to the corresponding author.

## Ethics Statement

The animal study was reviewed and approved by Animal Ethics Committee of Sichuan Agricultural University (Approval No. 2016-015).

## Author Contributions

QG and AC conceived and designed the experiments and wrote the manuscript. QG and SL performed the experiments. QG, AC, MW, RJ, SC, DZ, ML, XZ, QY, YW, SZ, JH, SM, XO, DS, and BT developed the methods. All authors read and approved the final manuscript.

## Conflict of Interest

The authors declare that the research was conducted in the absence of any commercial or financial relationships that could be construed as a potential conflict of interest.

## Publisher’s Note

All claims expressed in this article are solely those of the authors and do not necessarily represent those of their affiliated organizations, or those of the publisher, the editors and the reviewers. Any product that may be evaluated in this article, or claim that may be made by its manufacturer, is not guaranteed or endorsed by the publisher.

## References

[B1] Arambašic M. B. Randhawa M. A. (2014). Comparison of the methods of finney and miller-tainter for the calculation of LD50 values. *World Appl. Sci. J*. 32 2167–2170. 10.5829/idosi.wasj.2014.32.10.9132

[B2] Bulieris P. V. Behrens S. Holst O. Kleinschmidt J. H. (2003). Folding and insertion of the outer membrane protein OmpA is assisted by the chaperone Skp and by lipopolysaccharide. *J. Biol. Chem*. 278 9092–9099. 10.1074/jbc.M211177200 12509434

[B3] Caruana J. C. Walper S. A. (2020). Bacterial membrane vesicles as mediators of microbe - microbe and microbe - host community interactions. *Front. Microbiol*. 11:432–456. 10.3389/fmicb.2020.00432 32265873 PMC7105600

[B4] Chang F. F. Chen C. C. Wang S. H. Chen C. L. (2019). Epidemiology and antibiogram of *riemerella anatipestifer* isolated from waterfowl slaughterhouses in taiwan. *J. Vet. Res*. 63 79–86. 10.2478/jvetres-2019-0003 30989138 PMC6458550

[B5] Chu C. Y. Liu C. H. Liou J. J. Lee J. W. Cheng L. T. (2015). Development of a subunit vaccine containing recombinant *Riemerella anatipestifer* outer membrane protein A and CpG ODN adjuvant. *Vaccine* 33 92–99. 10.1016/j.vaccine.2014.11.010 25448104

[B6] Crasta K. C. Chua K. L. Subramaniam S. Frey J. Loh H. Tan H. M. (2002). Identification and Characterization of CAMP cohemolysin as a potential virulence factor of *Riemerella anatipestifer*. *J. Bacteriol*. 184 1932–1939. 10.1128/jb.184.7.1932-1939.2002 11889100 PMC134935

[B7] Gao Q. Tang T. Cheng A. C. Wang M. S. Jia R. Y. Zhu D. K. (2016). Development of an indirect ELISA using recombinant ompH protein for serological detection of *Riemerella anatipestifer* infection in ducks. *Int. J. Clin. Exp. Med*. 9 1330–1337.

[B8] Hatfaludi T. Hasani K. A. Boyce J. D. Adler B. (2010). Outer membrane proteins of *Pasteurella multocida*. *Vet. Microbiol*. 144:27. 10.1016/j.vetmic.2010.01.027 20197220

[B9] Holck A. Lossius I. Aasland R. Kleppe K. (1987). Purification and characterization of the 17 K protein, a DNA-binding protein from *Escherichia coli*. *Biochim. Biophys. Acta* 914 49–54. 3300779 10.1016/0167-4838(87)90160-9

[B10] Hu D. Guo Y. Q. Guo J. Wang Y. Pan Z. Xiao Y. C. (2019). Deletion of the *Riemerella anatipestife*r type IX secretion system gene sprA results in differential expression of outer membrane proteins and virulence. *Avian Pathol.* 48 191–203. 10.1080/03079457.2019.1566594 30640518

[B11] Hu Q. H. Ding C. Tu J. Wang X. L. Han X. G. Duan Y. B. (2012). Immunoproteomics analysis of whole cell bacterial proteins of *Riemerella anatipestifer*. *Vet. Microbiol*. 157 428–438. 10.1016/j.vetmic.2012.01.009 22317978

[B12] Hu Q. H. Han X. G. Zhou X. J. Ding C. Zhu Y. Y. Yu S. Q. (2011). OmpA is a virulence factor of *Riemerella anatipestifer*. *Vet. Microbiol*. 150 278–283. 10.1016/j.vetmic.2011.01.022 21349662

[B13] Hu Y. C. Cheng L. X. Zhong W. Chen M. H. Zhang Q. (2019). Bioinformatics analysis of gene expression profiles for risk prediction in patients with septic shock. *Med. Sci. Monit*. 25 9563–9571. 10.12659/MSM.918491 31838482 PMC6929537

[B14] Kang M. Seo H. S. Soh S. H. Jang H. K. (2018). Immunogenicity and safety of a live *Riemerella anatipestifer* vaccine and the contribution of IgA to protective efficacy in Pekin ducks. *Vet. Microbiol*. 222 132–138. 10.1016/j.vetmic.2018.07.010 30037633

[B15] Kim S. H. Jung D. I. Yang I. Y. Kim J. Lee K. Y. Nochi T. (2011). M cells expressing the complement C5a receptor are efficient targets for mucosal vaccine delivery. *Eur. J. Immunol*. 41 3219–3229. 10.1002/eji.201141592 21887786

[B16] Koski P. Rhen M. Kantele J. Vaara M. (1989). Isolation. cloning, and primary structure of a cationic 16-kda outer membrane protein of salmonella typhimurium. *J. Biol. Chem*. 264 18973–18980. 10.1016/s0021-9258(19)47253-0 2681205

[B17] Liao H. B. Cheng X. J. Zhu D. K. Wang M. S. Jia R. Y. Chen S. (2015). TonB energy transduction systems of riemerella anatipestifer are required for iron and hemin utilization. *PLoS One* 2015:127506. 10.1371/journal.pone.0127506 26017672 PMC4446302

[B18] Liu M. F. Wang M. Y. Zhu D. K. Wang M. S. Jia R. Y. Chen S. (2016). Investigation of TbfA in *Riemerella anatipestifer* using plasmid-based methods for gene over-expression and knockdown. *Sci. Rep*. 6 37159–37168. 10.1038/srep37159 27845444 PMC5109031

[B19] Liu M. F. Zhang L. Huang L. Biville F. Zhu D. K. Wang M. S. (2017). Use of natural transformation to establish an easy knockout method in *Riemerella anatipestifer*. *Appl. Environ. Microbiol*. 83 17–27. 10.1128/AEM.00127-17 28258143 PMC5394337

[B20] Lugtenberg B. Boxtel R. V. Evenberg D. Jong M. D. Storm P. Frik J. (1986). Biochemical and immunological characterization of cell surface proteins of *pasteurella multocida* strains causing atrophic rhinitis in swine. *Infect. Immun*. 52 175–183. 3957426 10.1128/iai.52.1.175-182.1986 PMC262216

[B21] Luo H. Y. Liu M. F. Wang L. Y. Zhou W. S. Wang M. S. Cheng A. C. (2015). Identification of ribosomal RNA methyltransferase gene ermF in *Riemerella anatipestifer*. *Avian Pathol*. 44 162–168. 10.1080/03079457.2015.1019828 25690020

[B22] Luo Y. G. Glisson J. R. Jackwood M. W. Hancock R. E. W. Bains M. Cheng I. H. (1997). Cloning and characterization of the major outer membrane protein gene (*ompH*) of *Pasteurella multocida* X-73. *J. Bacteriol.* 179 7856–7864. 9401047 10.1128/jb.179.24.7856-7864.1997 PMC179751

[B23] Madeira F. Park Y. M. Lee J. Buso N. Gur T. Madhusoodanan N. (2019). The EMBL-EBI search and sequence analysis tools APIs in 2019. *Nucleic Acids Res*. 47 W636–W641. 10.1093/nar/gkz268 30976793 PMC6602479

[B24] Mishra P. Singh U. Pandey C. M. Mishra P. Pandey G. (2019). Application of student’s t-test, analysis of variance, and covariance. *Ann. Card. Anaesth*. 22 407–411. 10.4103/aca.ACA_94_19 31621677 PMC6813708

[B25] Navidinia M. Soleimani N. Abadi N. B. (2019). Effect of recombinant *helicobacter* outer membrane protein H (HopH) on nitric oxide production by peripheral macrophage in BALB/c Mice. *Avicenna J. Med. Biotechnol*. 11 229–233. 31379995 PMC6626510

[B26] Osborn M. J. Munson R. (1974). Separation of the inner (cytoplasmic) and outer membranes of Gram-negative bacteria. *Methods Enzymol.* 31 642–653. 4608978 10.1016/0076-6879(74)31070-1

[B27] Pathanasophon P. Phuektes P. Tanticharoenyos T. Narongsak W. Sawada T. (2002). A potential new serotype of *Riemerella anatipestifer* isolated from ducks in Thailand. *Avian Pathol*. 31 267–270. 10.1080/03079450220136576 12396349

[B28] Pfaffl M. W. (2001). A new mathematical model for relative quantification in real-time RT-PCR. *Nucleic Acids Res*. 29 45–51. 10.1093/nar/29.9.e45 PMC55695 11328886

[B29] Schäfer U. Beck K. Müller M. (1999). Skp, a molecular chaperone of gram-negative bacteria, is required for the formation of soluble periplasmic intermediates of outer membrane proteins. *J. Biol.* Chem. 274 24567–24574. 10455120 10.1074/jbc.274.35.24567

[B30] Segers P. Mannheim W. Vancanneyt M. Brandt K. D. Hinz K. H. Kersters K. (1993). *Riemerella anatipestifer* gene nov. comb. nov., the causative agent of septicemia anserum exsudativa, and its phylogenetic affiliation within the *flavobacterium-cytophaga* rRNA Homology Group. *Int. J. Syst. Bacteriol*. 43 768–776. 8240957 10.1099/00207713-43-4-768

[B31] Slusky J. S. G. Dunbrack R. L. (2013). Charge asymmetry in the proteins of the outer membrane. *Bioinformatics* 29 2122–2128. 10.1093/bioinformatics/btt355 23782617 PMC3740626

[B32] Thanasarasakulpong A. Poolperm P. Tankaew P. Sawada T. Sthitmatee N. (2015). Protectivity conferred by immunization with intranasal recombinant outer membrane protein H from *Pasteurella multocida* serovar A:1 in chickens. *J. Vet. Med. Sci*. 77 321–326. 10.1292/jvms.14-0532 25650149 PMC4383778

[B33] Thein M. Sauer G. Paramasivam N. Grin I. Linke D. (2010). Efficient subfractionation of gram-negative bacteria for proteomics studies. *J. Proteome Res*. 9 6135–6147. 20932056 10.1021/pr1002438

[B34] Tian X. Huang L. Wang M. S. Biville F. Zhu D. K. Jia R. Y. (2020). The functional identification of Dps in oxidative stress resistance and virulence of *Riemerella anatipestifer* CH-1 using a new unmarked gene deletion strategy. *Vet. Microbiol*. 247 108730–108739. 10.1016/j.vetmic.2020.108730 32768200

[B35] Wang X. J. Liu W. B. Zhu D. K. Yang L. F. Liu M. F. Yin S. J. (2014). Comparative genomics of *Riemerella anatipestifer* reveals genetic diversity. *BMC Genomics* 15:479–489. 10.1186/1471-2164-15-479 24935762 PMC4103989

[B36] Wang Y. Tang C. Yu X. H. Xia M. Y. Yue H. (2010). Distribution of serotypes and virulence-associated genes in pathogenic *Escherichia coli* isolated from ducks. *Avian Pathol*. 39 297–302. 10.1080/03079457.2010.495742 20706886

[B37] Wang Y. Yin X. H. Zhou Z. T. Hu S. S. Li S. W. Liu M. (2019). Cas9 regulated gene expression and pathogenicity in *Riemerella anatipestifer*. *Microb. Pathog*. 136 103706–103714. 10.1016/j.micpath.2019.103706 31491547

[B38] Weiser J. N. Gotschlich E. C. (1991). Outer membrane protein A (OmpA) contributes to serum resistance and pathogenicity of *Escherichia coli* K-1. *Am. Soc. Microbiol.* 59 2252–2258. 10.1128/iai.59.7.2252-2258.1991 PMC258003 1646768

[B39] Xu X. X. Xu Y. H. Miao S. Jiang P. Cui J. S. Gong Y. S. (2020). Evaluation of the protective immunity of *Riemerella anatipestifer* OmpA. *Appl. Microbiol. Biotechnol*. 104 1273–1281. 10.1007/s00253-019-10294-3 31865436

[B40] Zhang X. Wang M. S. Liu M. F. Zhu D. K. Biville F. Jia R. Y. (2017). Contribution of RaeB, a Putative RND-Type transporter to aminoglycoside and detergent resistance in *Riemerella anatipestifer*. *Front. Microbiol*. 8:2435–2446. 10.3389/fmicb.2017.02435 29276505 PMC5727081

