# Supplementary material for: Putative Riemerella anatipestifer Outer Membrane Protein H Affects Virulence
Source: Front Microbiol. 2021 Sep 20;12:708225. doi: 10.3389/fmicb.2021.708225 (PMC8488386; doi:10.3389/fmicb.2021.708225)
Supplement: Supplementary file 1 [file Data_Sheet_1.docx]

Supplementary Material


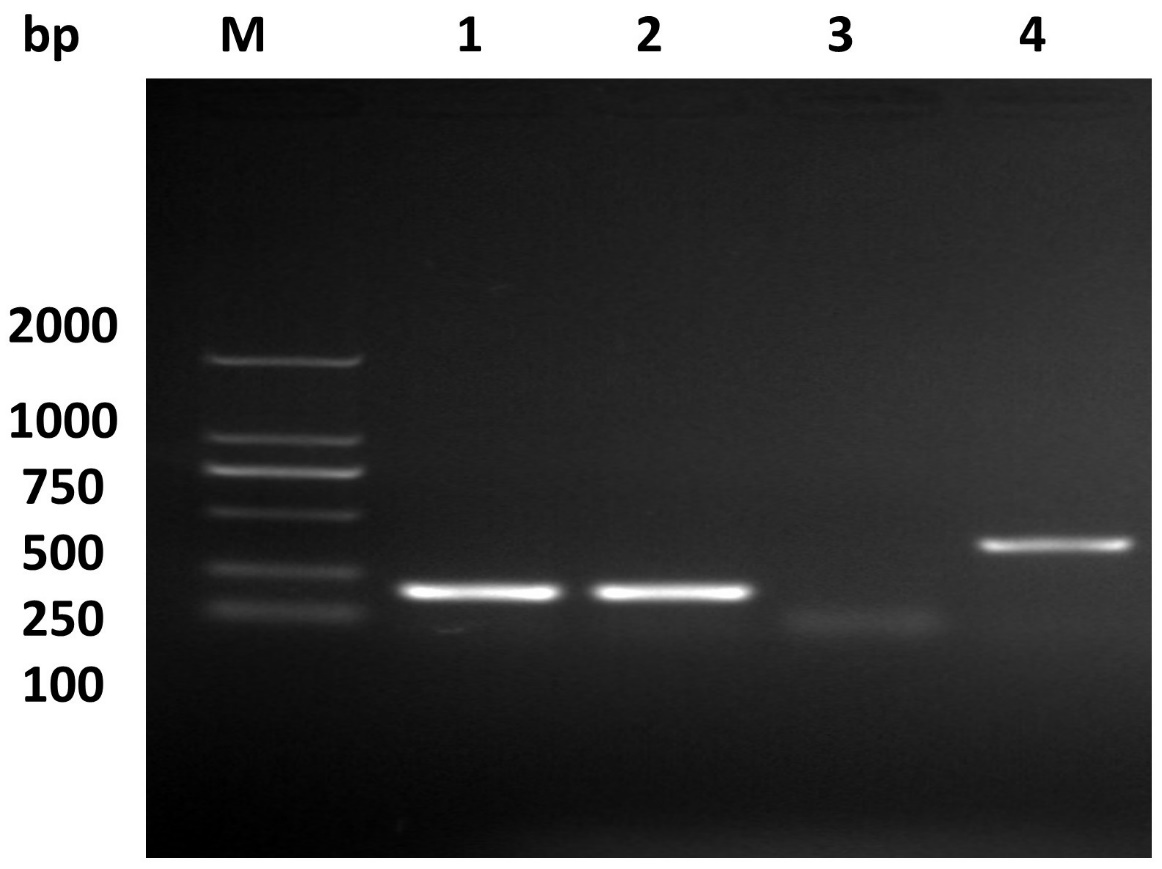


**Figure S1.** PCR confirmation of B739_0832 deletion in *R. anatipestifer*. Lanes 1 and 2 were PCR amplification of 16s RNA as positive control. Lanes 3 and 4 were PCR amplification of *B739_0832* gene from Δ*B739_0832* strain and wild-type, respectively. *B739_0832* gene is 501 bps long, and the primers pair with 50bps upstream and downstream of the gene.

A.


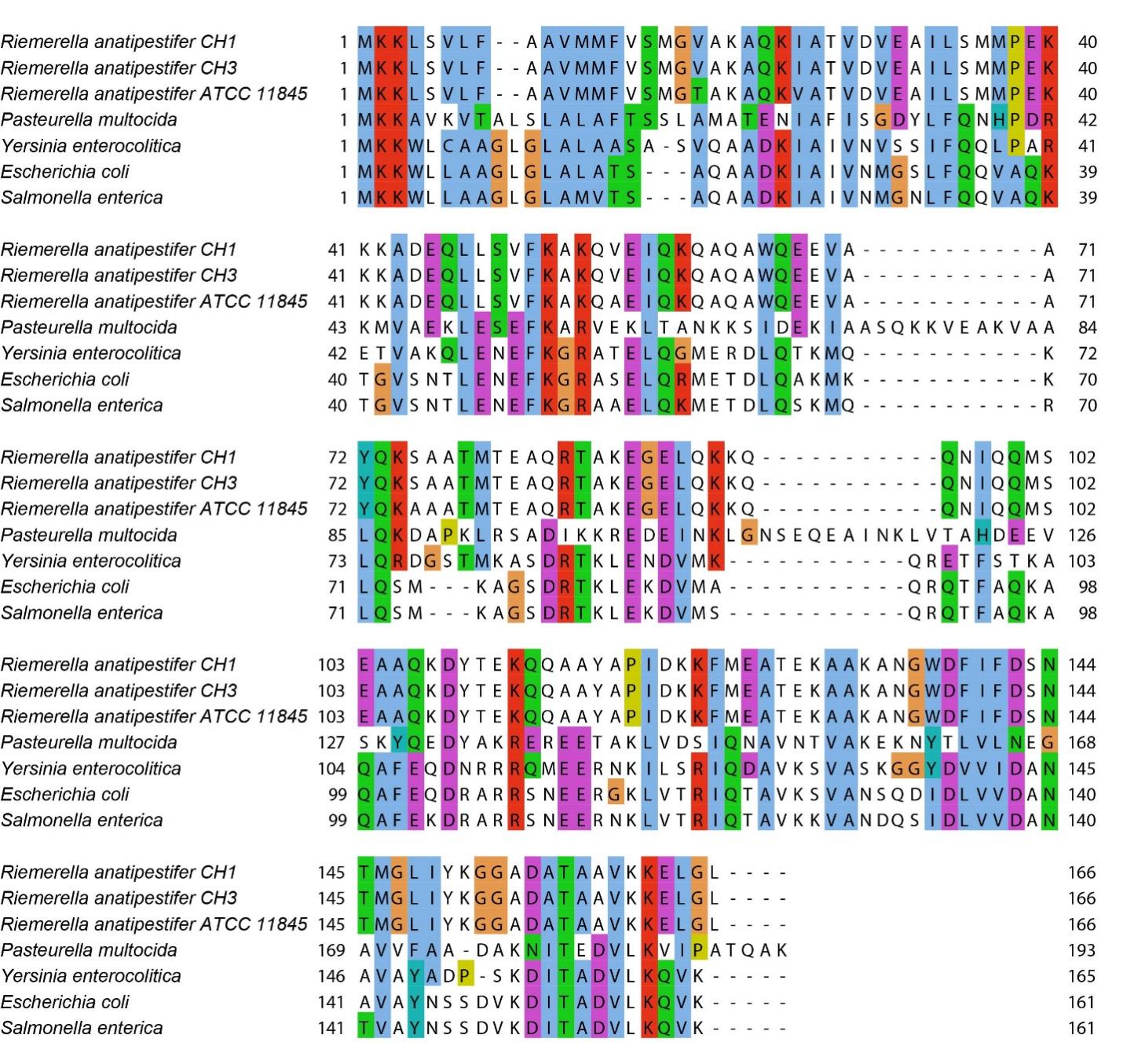


B.


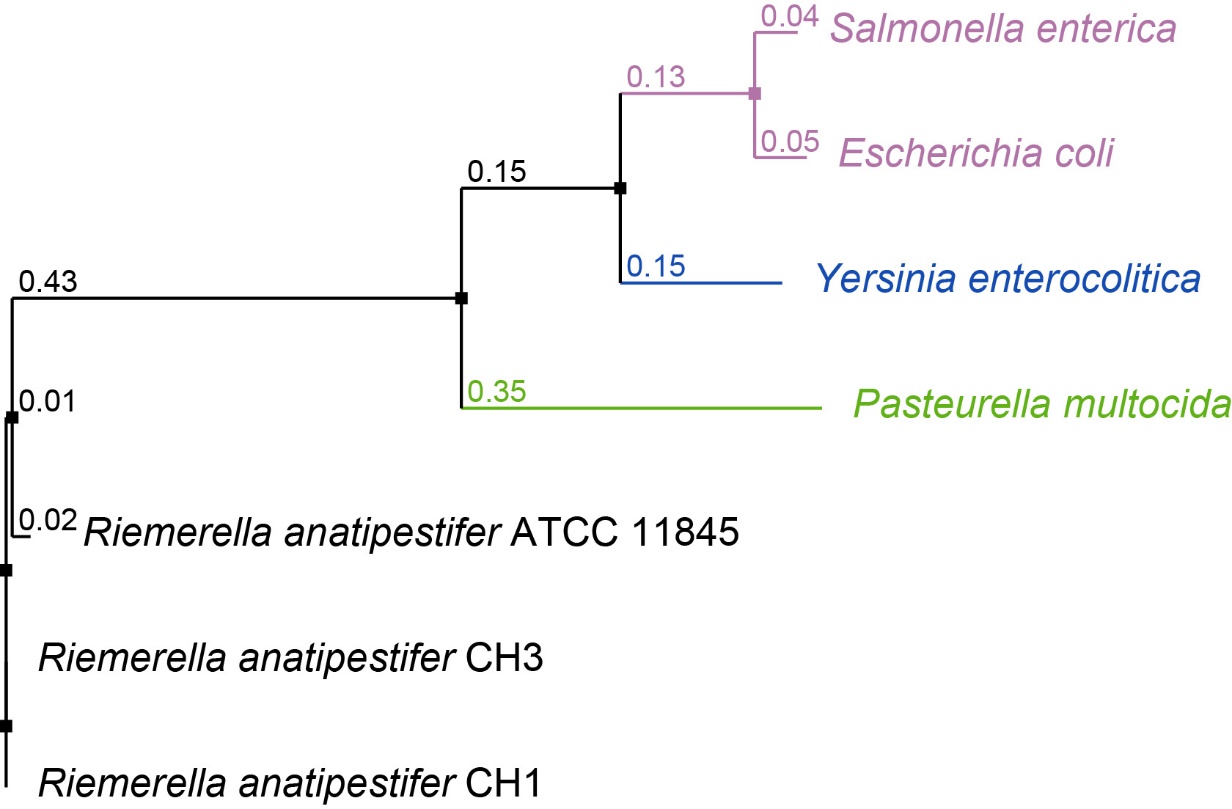


**Figure S2.** A. Sequence alignments of B739_0832 from *R. anatipestifer* CH-1, *R. anatipestifer* CH-2, *R. anatipestifer* ATCC, *E. coli*, *P. multocida*, *S. enterica* and *Y. enterocolitica* using EMBL-EBI Clustal Omega. B. Phylogenetic tree illustrating evolutionary distance among these OmpH/Skp proteins. Phylogenetic tree was also generated using EMBL-EBI Clustal Omega.


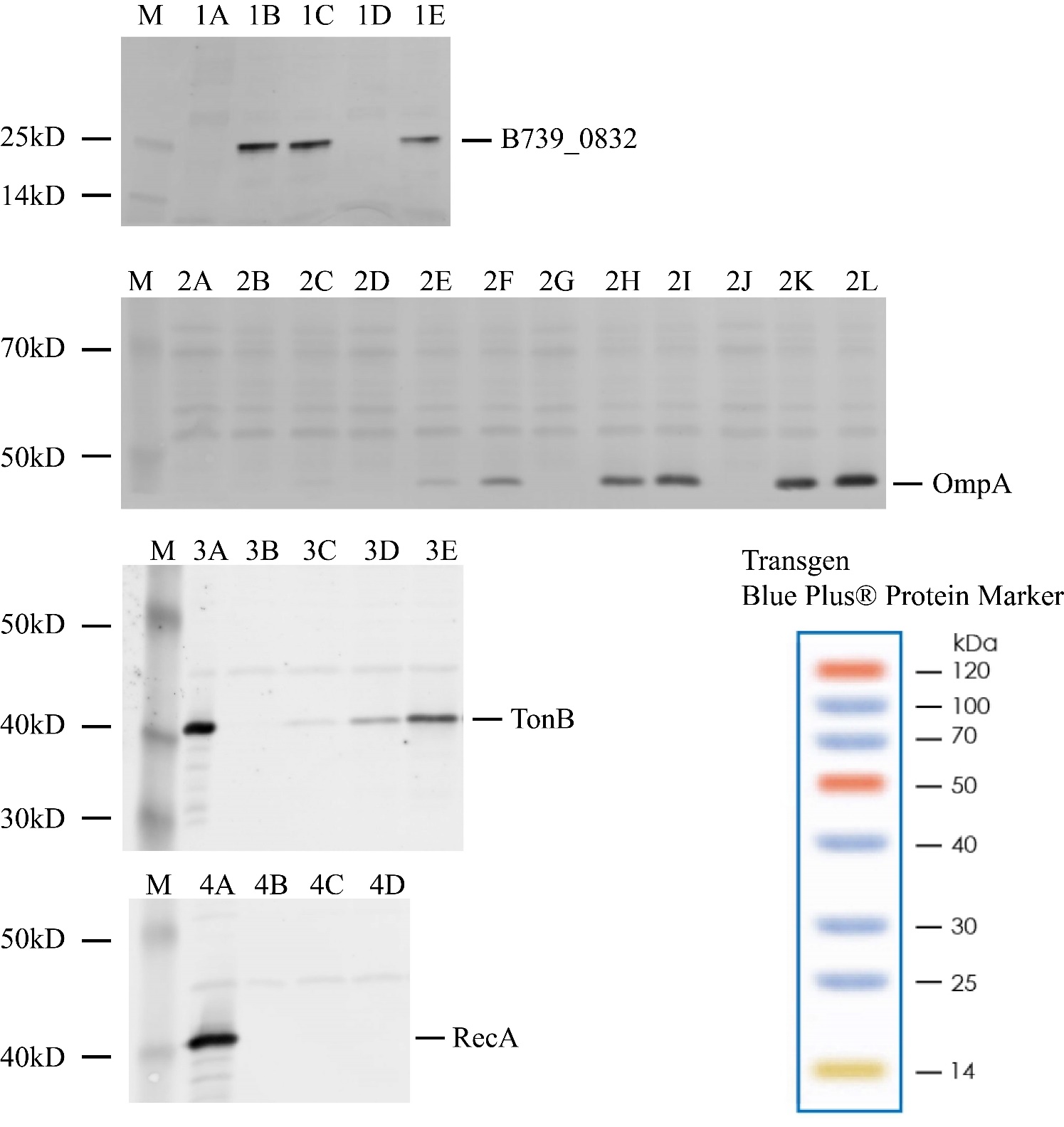


**Figure S3.** Membrane localization of *R*. *anatipestfer* CH-1 B739_0832 protein. Data correspond to Figure 2 in the main text with protein markers. Lane M is protein marker – Blue Plus® 14-120 kDa protein marker from Transgen (right bottom corner). Lanes 1A-1E are: inner membrane, whole cell, total membrane, inner membrane, and outer membrane samples, respectively. Lanes 2A-2L are: inner membrane, outer membrane, whole cell, total membrane, total membrane, whole cell, inner membrane, outer membrane, outer membrane, inner membrane, total membrane, and whole cell, respectively. Whereas 2A-2D were loaded with 1 µL sample in each lane; 2E-2H were loaded with 5 µL sample in each lane; and 2I-2L were loaded with 15 µL sample in each lane. Lanes 3A-3E are: TonB positive control sample, outer membrane, inner membrane, total membrane, and whole cell, respectively. Lanes 4A-4D are: whole cell, total membrane, inner membrane, and outer membrane, respectively. Protein sizes are approximation based on amino acid sequences.
